# A (RP)UHPLC/UV analytical method to quantify dsRNA during the mRNA vaccine manufacturing process†

**DOI:** 10.1039/d4ay00560k

**Published:** 2024-07-08

**Authors:** Sara Sousa Rosa, Shuran Zhang, Yustika Sari, Marco P. C. Marques

**Affiliations:** a Department of Biochemical Engineering, University College London Gordon Street London WC1E 6BT UK marco.marques@ucl.ac.uk; b Department of Bioengineering, iBB—Institute for Bioengineering and Biosciences, Instituto Superior Técnico, Universidade de Lisboa Lisboa Portugal; c Associate Laboratory i4HB—Institute for Health and Bioeconomy, Instituto Superior Técnico, Universidade de Lisboa Lisboa Portugal

## Abstract

dsRNA is a product related impurity produced during the mRNA manufacturing process. The established immuno-based detection methods lack the flexibility and speed required to be applied throughout the manufacturing process. The RP-HPLC method developed outperforms these in terms of precision, broader detection range, LOD and LOQ, as well as in output variance. Using this method, dsRNA can be quantified in under 30 min for a single sample.

## Abstract 2

Development of an HPLC method to quantify dsRNA, a product related impurity, produced during mRNA vaccine manufacturing. This method allows to quantify dsRNA in single samples in under 30 min.

## Graphical abstract

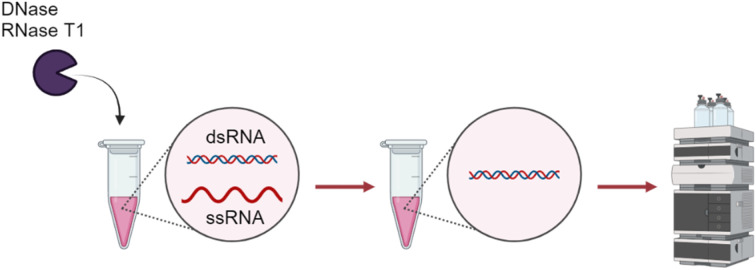

## Introduction

There has been widespread interest in mRNA vaccines within both academia and industry since the COVID-19 pandemic. Today, there are over 60 ongoing clinical trials that use mRNA vaccines for a variety of treatments that include prophylactic and cancer treatments, protein replacement and gene editing.^1^ When compared with traditional vaccine platforms, mRNA vaccines present a number of advantages, namely the fast production, the safety profile, and the vaccine effectiveness. From a clinical and manufacturing point-of-view, consistency of high-quality products is required with little batch-to-batch variability. This can only be achieved with a precise product characterisation and a tight manufacturing process control. Therefore, analytical procedures that rigorously characterise the mRNA and impurities (process- and product-related impurities)^2^ throughout the manufacturing process are required.^3^

mRNA vaccines are usually produced in a cell-free system where a linearised DNA strand is *in vitro* transcribed (IVT) into a mRNA strand. RNA polymerases (*e.g.* T7 RNA polymerase) transcribe the DNA with nucleotides as co-substrates to produce grams per litre of mRNA.^4–7^ Other reaction components include co-factors, enhancers and additional enzymes, with temperature and pH as critical reaction parameters. The exploration of different reaction conditions coupled with a tight production characterisation^8^ led already to an improvement in reaction yields, with reported production in the range of 12 g L^−1^.^7,9^ However, despite the tight control, there are impurities produced throughout the IVT process. These can be classified into process related (enzymes, residual NTPs, or the DNA template) and product related impurities (malformed mRNAs).^2,7^ During the IVT reaction, the T7 RNA polymerase can release truncated mRNA molecules, or produce complementary RNA strands that can hybridise and produce double-stranded mRNA (dsRNA). The presence of this particular impurity in the final product must be avoided since it can impact translation and trigger a strong immune response,^10^ which ultimately can lead to an uncontrolled immune-inflammatory reaction.^11^ In the absence of dsRNA, protein expression within cells can be increased by 10–1000 fold.^12^ Therefore, optimised reaction conditions or new purification methods are necessary to eliminate dsRNA as well as precise analytical procedures to quantify this impurity. Nevertheless, there is still a lack of defined concentration limits that are established by the regulatory agencies for dsRNA.^3^

From a reaction optimisation perspective, multiple approaches have been followed to reduce dsRNA such as engineering T7 RNA polymerase,^13,14^ or blocking the 3′ end with complementary oligonucleotides to avoid overextension.^15^ New chromatographic modalities can be applied, exploring physico-chemical differences between mRNA and dsRNA.^16^ Nonetheless, dsRNA needs to be carefully monitored during the manufacturing process itself and in the final product.^3^ Analytically, dsRNA can be detected and characterised by several methods, including electrophoresis,^17,18^ immunoassays that use anti-double-stranded RNA antibodies such as dot blot, ELISA or lateral flow strip assay (LFSA),^19^ and asymmetric flow field flow fractionation (A4F),^20^ or even chromatographic methods.^8,21–23^ Nevertheless, there is a lack of well-established methods that can be applied in the manufacturing process which will quantify dsRNA that meet the regulatory requirements,^24^ are quick, specific and minimise impurity interference, and can be easily adapted to the different process stages, from the production to the different purification steps, and to the different modalities of the mRNA process (batch, fed-batch, or continuous).

In this communication, we explore a method to quantify dsRNA in the different stages of the manufacturing process. A previously established reverse-phase HPLC method that quantifies total mRNA was coupled to an enzymatic digestion step that digests ssRNA, allowing us to directly measure dsRNA. The developed RP-HPLC method achieved lower limit of detection (LOD) and limit of quantification (LOQ), 1.41 × 10^−2^ ± 1.78 × 10^−3^ g L^−1^, and 4.27 × 10^−2^ ± 5.4 × 10^−3^ g L^−1^, respectively, compared to the current golden standard, the dot blot. Furthermore, the decision limit (CC*α*, 9.95 × 10^−2^ ± 1.26 × 10^−3^ g L^−1^) and detection capability (CC*β*, 1.70 × 10^−2^ ± 2.14 × 10^−3^ g L^−1^) are low, and obtained for a single sample under 30 min when compared with standard methods (*e.g.* ELISA and dot blot assay).^3^ HPLC outperformed these and proved to be precise, and less prone to operational errors, even in spiking studies with process-related impurities (NTPs and DNA) where minimal variance in quantification is observed. The implemented RP-HPLC is robust to use during the mRNA manufacturing process and that can be adapted for Process Analytical Technology (PAT) purposes in the future.

## Materials and methods

Unless otherwise stated, all chemicals and reagents were purchased from Thermo Fisher Scientific (UK).

### Template plasmid DNA production

Template design and plasmid production was performed as previously described.^7^ Briefly, GFP gene (GenBank Accession # AAB02572.1) is flanked by 5′UTR containing the T7 RNA polymerase promoter, eukaryotic translation factor binding site and a Kozak consensus sequence,^25^ and by a 3′UTR composed of two β-globin tandem repeats followed by a 120 bp poly-A tail segmented with a 6 bp.^26^ A pUC7 containing a kanamycin resistance is used as a plasmid vector with plasmid propagation performed in *e. coli* NEB 10-beta (New England Biolabs, UK). The pDNA is obtained by performing an overnight culture in LB media at 37 °C and purified using the GeneJET Plasmid Miniprep Kit.

### Template production by touchdown polymerase chain reaction

The forward and reverse primers were previously described.^7^ A T7 RNA polymerase promoter sequence was added to the 5′ end of the complementary strand using the reverse primer. The DNA template for mRNA IVT is produced by touchdown PCR (Applied Biosystems™ Veriti™ 96-Well Thermal Cycler, Thermo Fisher Scientific, UK). Briefly, the reaction mixture comprises 250 ng mL^−1^ of template plasmid, 0.4 μM of forward and reverse primer, 1× VeriFi™ Buffer, 1× VeriMax Enhancer, and 0.02 U μL^−1^ high-fidelity VeriFi™ DNA polymerase (PCR Biosystems, UK). The PCR conditions for the denaturation step are 98 °C for 30 s, 20 cycles with a denaturation step at 94 °C for 15 s, an annealing step at 65–55 °C for 30 s and an extension step at 72 °C for 45 s. This is followed by 20 cycles of an annealing step at 55 °C for 30 s and an extension step at 72 °C. The final extension is performed at 72 °C for 2 min. The obtained PCR product is purified and concentrated using a GeneJET PCR Purification Kit. Final concentrations are quantified on a NanoDrop™ One Microvolume UV-Vis Spectrophotometer (Thermo Fisher Scientific, UK).

### dsRNA *in vitro* transcription reactions

dsRNA is produced in an IVT reaction using previously described reaction conditions.^7^ Briefly, 89 nM DNA template is mixed with 7.7 mM NTPs, 5.3 mM dithiothreitol, 50 mM magnesium acetate, 40 mM Tris–HCl pH 6.8, 2.3 mM spermidine, 8 U mL^−1^ inorganic pyrophosphatase and 7750 U mL^−1^ of T7 RNA polymerase. To this mix, 1500 U mL^−1^ RNase inhibitor is added to avoid degradation. The mixture is incubated at 43 °C for two hours on an Applied Biosystems™ Veriti™ 96-Well Thermal Cycler (Thermo Fisher Scientific, UK). After incubation, the produced strands of RNA are annealed to form dsRNA by diluting the reaction mixture twice with WFI and incubating in a decreasing temperature gradient (from 85 °C to 30 °C) with a 2 min step at each temperature.

### dsRNA purification

The DNA template and the remaining mRNA are digested using TURBO™ DNase, and RNase T1, respectively. The enzymes are added to the IVT samples to a final concentration of 0.04 U μL^−1^ (TURBO™ DNase), and 20 U μL^−1^ (RNase T1). The samples are incubated at 37 °C for 30 min and purified afterwards using a MEGAclear™ Transcription Clean-Up kit. After purification, the dsRNA is precipitated overnight at −20 °C by adding 500 mM pH 5 ammonium acetate and 2.5 volumes of ethanol. The samples are centrifuged at 15 000 × *g* for 15 min and the supernatant was discarded. The pellet is air dried and WFI water is added to resuspend the pellet to the desired final concentration. Concentration is determined on a NanoDrop™ One Microvolume UV-Vis Spectrophotometer (Thermo Fisher Scientific, UK) using a conversion factor of 47 μg mL^−1^ Abs_260_^−1^.^27^

### Analytical methodologies

#### Gel electrophoresis

Samples obtained from the IVT were digested with T1 RNAse as previously described and analysed by gel electrophoresis.^7^ Briefly, a 2% (w/v) agarose gel was prepared with 0.5× TBE buffer with 5.5 mM magnesium chloride and prestained with SYBR® Safe DNA Gel Stain and run at 100 V for one hour. A 1 kb Plus DNA Ladder (New England Biolabs, UK) was used for analysis.

The samples were blotted onto a 0.45 μm nitrocellulose membrane using a pipette tip. The membrane was dried and incubated with 3% (w/v) of BSA (Sigma-Aldrich, USA) in 1× PBS–Tween buffer, containing 1× PBS (10 mM sodium phosphate, 2.68 mM potassium chloride, and 140 mM calcium chloride) and 0.05% (v/v) of Tween-20 (Sigma-Aldrich, USA), for an hour at 22 °C. The membrane was subsequently incubated for an hour with 1 : 1500 dilution of SCICONS™ J2 mouse anti-dsRNA IgG2a monoclonal antibody (Nordic-MUbio, Netherlands) as primary antibody using 3% (w/v) BSA in 1× PBS–Tween buffer. The membrane was washed by incubating for 5 min in the PBS–Tween buffer and repeated three times. Afterwards, the membrane was incubated with 1 : 2000 dilution of secondary antibody, mouse IgG horseradish peroxidase (HRP)-conjugated antibody (R&D Systems, UK) for an hour, followed by three washing steps. Prior to the chemiluminescence exposure, the membrane was treated with the Pierce™ ECL western blotting substrate and incubated for 3 min. All the incubation steps were performed at 22 °C. The visualisation was performed through chemiluminescence exposure using an Amersham™ Imager 600 (GE Healthcare, UK).

#### Reverse-phase high performance liquid chromatography

HPLC analysis^7,28^ was performed on an UltiMate 3000 UHPLC System (Thermo Fisher Scientific, UK) equipped with a VWD-3400 RS Rapid Detector. All species were analysed on a RP-DNApac column (2.1 × 100 nm, Thermo Fisher Scientific, UK) at 80 °C with detection at 260 nm. The column was pre-equilibrated with TAE buffer (100 mM Tris acetate, pH 7, 2.5 mM EDTA), with the initial flow rate set to 0.2 mL min^−1^. After a 1 min washing step, the flow rate was increased to 0.35 mL min^−1^, at a gradient of 0.25 m min^−1^ gradient over 30 s. A first elution gradient is performed to 6% of the elution buffer (TAE buffer, 25% acetonitrile) for 30 s at 0.35 mL min^−1^, followed by a gradient of 0.4 mL min^−1^ over 4 min until 76.5% elution buffer is reached, finalising with gradient to 100% elution buffer for 1 min. The column is washed with the elution buffer for 3 min and re-equilibrated with TAE buffer for 6 min at 0.4 mL min^−1^.

#### Enzyme-linked immunosorbent assay

The SCICONS anti-dsRNA ELISA kit, J2 based (Nordic MUBio, NL) was used to detect dsRNA. Briefly, 96 well plates were treated with anti-dsRNA coating antibody diluted in PBS and incubated overnight at 4 °C. The plates were then incubated with 1% BSA in PBS for 1 h at 37 °C and were washed with PBS–T (1× PBS with 0.5% Tween 20) three times. 100 μL of samples were added to each well and incubated for 1 hour at 37 °C. Afterwards, the plates were washed 4 times with PBS–T and incubated with the anti-dsRNA antibody at 37 °C for 1 hour. After another washing step, HRP-conjugated goat-anti mouse secondary antibody diluted in PBS with 1% BSA is added and incubated at 37 °C for 1 hour. The plates are washed a final time, and the TMB substrate is added. The plates are incubated for 15 to 30 min at 22 °C. The reaction is stopped by adding H_2_SO_4_ to a final concentration of 1 M. The absorbance is read at 450 nm.

#### Spiking studies

Spiking studies were performed by mixing dsRNA with DNA or NTPs (Table 1). RNA without any spiking components, isolated NTPs and DNA and the mixture of both impurities were used as controls. The dsRNA samples were quantified using HPLC and dot blot methods.

**Table 1:** Concentration of dsRNA DNA and NTP concentrations used for the spiking studies

Species	Concentration	
	Min.	Max.
RNA (g L^−1^)	0.1	0.4
DNA (nM)	10	90
NTPs (mM)	2	7.5

#### Data and statistical analysis

All the statistical analyses were performed in *R*. At least three independent experiments and three dependent measures were performed for each experiment, and all the data are presented as mean ± standard deviation. One-way ANOVA followed by Tukey's test were used for analysis. A *p* value < 0.05 was considered to indicate statistical significance. The normality plot of the residuals can be found in ESI Fig. S1 and S2,† for the calibration curves and dsRNA spiking studies, respectively. The Shapiro–Wilk test of the ANOVA residuals was performed, and no violation of normality was detected.

#### Range validation

The limits of detection (LOD) and quantification (LOQ) were calculated based on the standard error of the intercept (*σ*) and the slope (*S*) of the calibration curves at a signal-to-noise ratio of 3.3 (LOD) and 10 (LOQ),^29^ according to

1 LOD = 3.3*σ*/*S*

2 LOQ = 10*σ*/*S*

where *S* and *σ* are the slope and standard deviation of the response, respectively. The decision limit (CC*α*) and detection capability (CC*β*) were calculated considering a 2.33 factor, which corresponds to 1% of false positive risk, and a 1.64 factor, which corresponds to a 5% false negative risk with regard to CC*α*,^24,30^ according to

3 CC*α* = 2.33*σ*/*S*

4 CC*β* = CC*α* + 1.64*σ*/*S*

## Results and discussion

We have explored reverse phase chromatography (RP-HPLC) for the detection of dsRNA based on the physico-chemical properties of mRNA, namely the hydrophobicity.^31^ Preparative chromatography, namely ion-pair (IP-RP-HPLC), has already been used to separate ssRNA from dsRNA.^12,32^ The ion-pairs added to the mobile phase will interact with the negatively charged backbone of oligonucleotides and will allow separation according to the number of backbones available.^33^ In this work, the mobile phase is composed of Tris buffer and EDTA, instead of an ion-pair and thus the separation is achieved by the hydrophobic portions present in the mRNA (*e.g.* poly(A) tail).^28^ To facilitate detection, it is necessary to ensure that solely dsRNA is present in samples. To achieve this, an enzymatic digestion prior to injection is performed using RNase T1.^7,13^ The RNase T1 is an endoribonuclease that cleaves single-stranded RNA at the guanosine residues. When added to an IVT reaction, RNase T1 will digest all the ssRNA, and the dsRNA can be directly quantified by RNA quantification methods. The semi-quantitative assays such as the dot blot or ELISA can be performed without the use of this additional enzymatic digestion since they use specific antibodies (*e.g.* dsRNA antibody j2).^34^ However, with these assays, no direct read out of the samples is possible and multiple assay preparation steps are often required, which can potentially increase the associated operator error.

**Fig. 1:**
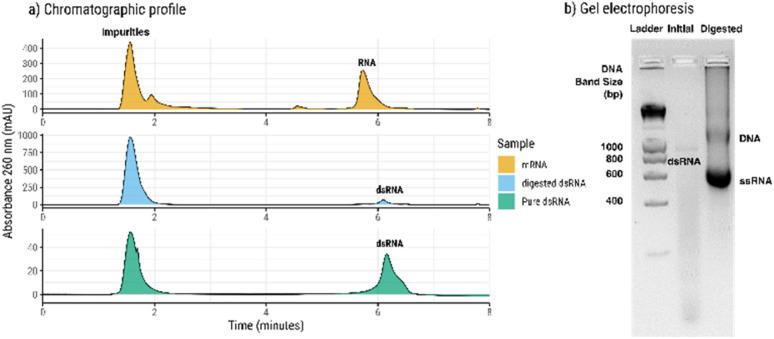
Analysis of dsRNA by RP-HPLC and corresponding gel electrophoresis of the samples. (a) Chromatographic profile of total mRNA (yellow) and dsRNA before (blue) and after purification (green) obtained by the developed RP-HPLC method. (b) Agarose gel electrophoresis of the total mRNA (initial) and after digestion with RNase T1 (digested).

The RP-HPLC separates molecules according to its hydrophobicity (Fig. 1a). Small size impurities (*e.g.* reaction components) do not usually bind to the stationary phase and are eluted during the wash phase (first peak in the chromatograms), while mRNA is eluted during the gradient phase. This is achieved by increasing the organic solvent concentration in the mobile phase. After digestion with the RNase T1 enzyme, the digested ssRNA is eluted during the wash phase while the dsRNA is bound to the stationary phase and requires organic solvent to be eluted. After digestion, there is a shift in the retention time of the RNA peak. This can be attributed to the difference in the molecular weight between dsRNA and ssRNA (Fig. 1b). The dsRNA produced during IVT will have single stranded regions, possible interacting with the stationary phase, while after digestion, only double stranded regions are present.

**Fig. 2:**
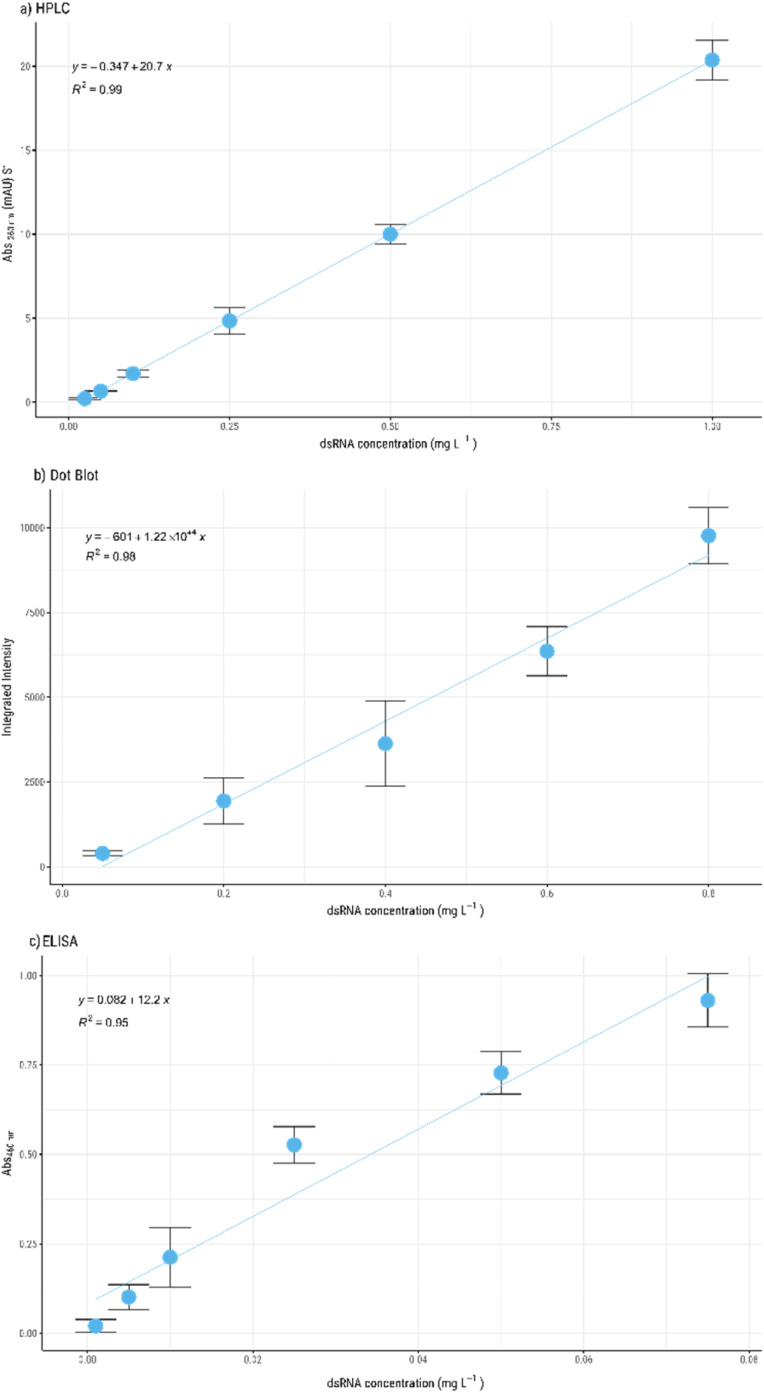
dsRNA calibration curves and corresponding linear regression obtained for the analytical method used: HPLC (a), dot blot (b) and ELISA (c). Three independent linear regression analyses were performed, and 3 independent samples were analysed.

We have validated the HPLC method by analysing the different regulatory requirements (*e.g.* specificity, range, accuracy, precision, and/or robustness^24^) and compare it to dot blot and commercially available dsRNA detection ELISA kits (Fig. 2). To achieve this, 6-point calibration curves were prepared with dsRNA concentrations ranging from 0.025 to 1 g L^−1^, 0.05 to 1 g L^−1^ and 0.005 to 0.075 mg L^−1^, for the HPLC, dot blot and ELISA, respectively. The standard curves were evaluated using three independent samples. In the HPLC method, linearity is observed throughout the range of sample concentrations used with a high correlation coefficient, *R*^2^ > 0.99 (Fig. 2a). In contrast, the dot blot only presents a linear relationship at concentrations below 0.8 g L^−1^ with *R*^2^ > 0.98 (Fig. 2b). However, the residual evaluation data show that there is homoscedasticity (ESI, Fig. S1.b†), which means that although the *R*^2^ is lower, the variance between samples is similar. The ELISA method is most specific of the methods evaluated, as it can detect the smallest concentration of dsRNA. However, this assay has a very narrow range of detection. Linearity is only achieved at concentration below 0.07 mg L^−1^ (Fig. 2c) with a low correlation coefficient, *R*^2^ > 0.95. The lower correlation can be attributed to the high sensitivity of the method which makes the process more prone to operator errors. The residual analysis (ESI, Fig. S1†) and the respective model coefficient (*p*-value) evaluation show that there is a strong correlation between the variables for the three methods, confirming the goodness of fit of the resulting models.

The limit of detection (LOD) and limit of quantification (LOQ) were calculated for the three methods (Table 2). ELISA presents the lowest LOD and LOQ, 1.51 × 10^−5^ ± 3.21 × 10^−6^ and 4.56 × 10^−5^ ± 9.73 × 10^−6^ g L^−1^, respectively. The dot blot presents the highest LOD (0.201 ± 0.06 g L^−1^) and LOQ (0.609 ± 0.181 g L^−1^) whilst the HPLC presents 14 times lower LOD and LOQ (0.014 ± 0.002, 0.043 ± 0.005 g L^−1^), when compared with dot blot assay.

**Table 2:** Range characterisation of the three dsRNA quantification methods evaluated (HPLC, dot blot and ELISA) in terms of limit of detection (LOD), limit of quantification (LOQ), decision limit (CC*α*) and the detection capability (CC*β*)

Parameter	Analytical method					
	HPLC		Dot blot		ELISA	
	*x̄* (g L^−1^)	*σ* (g L^−1^)	*x̄* (g L^−1^)	*σ* (g L^−1^)	*x̄* (g L^−1^)	*σ* (g L^−1^)
LOD	1.41 × 10^−2^	1.78 × 10^−3^	2.01 × 10^−1^	5.96 × 10^−2^	1.51 × 10^−5^	3.21 × 10^−6^
LOQ	4.27 × 10^−2^	5.40 × 10^−3^	6.09 × 10^−1^	1.81 × 10^−1^	4.56 × 10^−5^	9.73 × 10^−6^
CC*α*	9.95 × 10^−3^	1.26 × 10^−3^	1.42 × 10^−1^	4.21 × 10^−2^	1.06 × 10^−5^	2.27 × 10^−6^
CC*β*	1.70 × 10^−2^	2.14 × 10^−3^	2.42 × 10^−1^	7.17 × 10^−2^	1.81 × 10^−5^	3.86 × 10^−6^

The decision limit (CC*α*) and detection capability (CC*β*) were evaluated for all methods. CC*α* represents the lowest concentration obtained which is reliable (1% of false positive risk), while CC*β* is the lowest concentration possible to measure with an error probability of 5% (false negative risk).

ELISA assay presents the lowest CC*α* and CC*β* values (1.06 × 10^−5^ ± 2.27 × 10^−6^ and 1.81 × 10^−5^ ± 3.86 × 10^−6^ g L^−1^) while in contrast, dot blot presents the highest values (0.142 ± 0.042 and 0.242 ± 0.07 g L^−1^). Since ELISA presents the lowest LOD and LOQ evaluated, it can be an ideal method to use with highly pure samples with residual amounts of impurities, namely dsRNA. Currently approved vaccines control the dsRNA levels to be as low as possible throughout the manufacturing process^35,36^ with no defined value. Currently, the dot blot assay is the method recommended to characterise dsRNA throughout the mRNA manufacturing process^3^ and evaluation values obtained support this. Nevertheless, the developed HPLC method shows a larger detection range and limits, presenting itself as a more versatile quantification method. Additionally, these ranges can be extended as the maximum concentration of dsRNA that can be quantified by HPLC is contingent on the saturation capacity of the column itself, which can be adapted by selecting columns of different diameters and lengths.

**Fig. 3:**
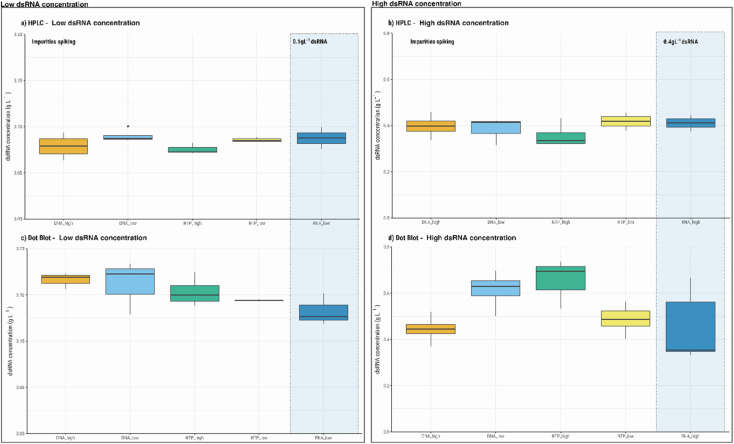
Spiking studies and respective distribution of dsRNA concentration obtained using HPLC and dot blot. HPLC results using 0.1 g L^−1^ (a) and 0.4 g L^−1^ of dsRNA (b). Dot blot results using 0.4 g L^−1^ (c) and 0.2 g L^−1^ of dsRNA (d). DNA_high and DNA_low correspond to a spiking with 90 and 10 nM of DNA template, respectively. NTP_low and NTP_high correspond to 2 and 7.5 mM, respectively. Blue shades areas correspond to the samples without spiking. At least 3 independent samples were analysed for each condition.

To evaluate further the accuracy and precision of the HPLC method implemented, a spiking study was performed. Pure dsRNA samples were spiked with two process related impurities that typically can be present in process samples, namely NTPs and the DNA template (Table 1). The concentrations of the impurities tested are in the range commonly encountered in the manufacturing process of mRNA vaccines. Additional controls with the spiking reagents (DNA and NTPs) and their mixtures with different concentrations evaluated were also performed. The HPLC precision performance was compared with that of dot blot assay as a similar range can be used in both methods. HPLC analysis shows that the values measured have a more uniform distribution when compared with the dot blot assay (Fig. 2). Statistical analysis performed shows that a significant difference is observed in the dot blot performed with a high concentration of dsRNA (Fig. 3d, 0.4 g L^−1^), and in the presence of a high concentration of NTPs (ESI, Tables S1–S4†). This may be due to the occurrence of unspecific binding of the antibody used to perform the dot blot assay.

No significant differences in the dsRNA measured concentrations were found when using HPLC, indicating that the method is more accurate (ESI, Tables S1–S4†). Comparing the concentrations obtained for high and low range of dsRNA concentrations, the results show that there are no significant differences between both methods. For high concentration of dsRNA (0.4 g L^−1^), HPLC measured 0.41 ± 0.05 g L^−1^, while with dot blot assay, a concentration of 0.45 ± 0.151 g L^−1^ was obtained. For low dsRNA concentrations (0.1 g L^−1^), 0.09 ± 0.016 g L^−1^ and 0.13 ± 0.017 g L^−1^ were obtained for the HPLC and dot blot assay, respectively.

Overall, the HPLC method presents a better precision and range, as it presented a better correlation between the concentration range (0.025 to 1 g L^−1^) evaluated when compared with dot blot assay, but ELISA presents the lowest detection range (0.005 to 0.075 mg L^−1^). Nevertheless, when comparing analytical methods, it is also required to look into the overall analytical performance (Table 3). Although ELISA is a method that can accurately measure the smallest concentration of dsRNA present in samples, the results are solely obtained after 12 hours due to the multiple incubation steps involved. Additionally, the multiple steps involved in this assay also increase operator error occurrence. In terms of time-to-result, the dot blot is the faster method among the immunoassays (three hours). Nonetheless, similar to ELISA, it requires multiple incubation steps and reagent additions. The HPLC is the fastest for single sample, requiring minimum sample preparation and operator input to run the method. However, the process time can increase if an additional digestion step with RNase T1 is required. Nevertheless, the throughput is limited to HPLC sampler capacity and the method running time. Higher throughputs are obtained with the ELISA and dot blot. On the other hand, the HPLC method is a fully quantitative method while dot blot relies on a semi quantitative analysis based on densitometry measurements.

**Table 3:** Summary of the method validation and performance overview of the three analytical methods evaluated for the measurement of dsRNA concentration in a single sample

	Method validation			Performance overview			
	Sensibility	Range	Precision	Assay time	Complexity	Operator error	Detection
ELISA	+++	+	n/a	>12 h	++	+++	Quantitative
Dot blot	++	++	++	>3 h	+	++	Semi-quantitative
HPLC	++	+++	+++	<1 h	+++	+	Quantitative

Cost can also be a predominant factor when choosing the analytical method to be used. By nature of the assay itself, the immuno-assays are costly. In particular, the dot blot costs are associated with the primary antibodies used. Comparatively, these are up to 10× higher than the RNase T1 enzyme required for HPLC assay, but cost will be reduced with increased throughput. Evidently, HPLC costs are strongly correlated with the stationary phase and equipment used. Finally, at the manufacturing level, HPLC can be used as an at-line method integrated in the process workflow^37^ allowing data to be obtained faster and consequently enabling a tight process control.

## Conclusions

dsRNA is a product related impurity produced during the mRNA vaccine manufacturing. This impurity has a strong impact on mRNA performance within the cells as it decreases the translation rate and increases the inflammatory response, and the presence of this particular impurity in the final product must be quantified and avoided.

In this work, the use of HPLC for dsRNA quantification is explored, and compared with established immunoassays, namely the dot blot and ELISA. With the developed RP-HPLC method it was possible to quantify dsRNA in samples in under 30 min. The sensitivity and precision of this method are high, with a broader detection range 0.025 to 1 g L^−1^ and minimum impurity detection interference. From a regulatory perspective, it achieved the lowest limit of detection (LOD) and limit of quantification (LOQ), 1.41 × 10^−2^ ± 1.78 × 10^−3^ g L^−1^, and 4.27 × 10^−2^ ± 5.4 × 10^−3^ g L^−1^, respectively, compared to the current golden standard, the dot blot. The occurrence of false positive or negatives with this method is also low given the decision limit and detection capability obtained.

Additionally, the implementation of the HPLC method requires minimum operator input and sample handling with throughput achieved by tunning the method running time. This, combined with the ability to use this method at-line and prone to automation makes the HPLC an ideal method to be used to quantify dsRNA throughout the manufacturing process. Precise and reliable analytical assays are of paramount importance to have a well-established manufacturing process that delivers high quality products.

## Data availability

The data supporting this article have been included as part of the ESI.† Raw data are available upon request from the authors.

## Author contributions

Conceptualization, M. P. C. M. and S. S. R.; data curation, formal analysis, investigation and methodology, S. S. R., S. Z., and Y. S.; supervision, M. P. C. M; validation, S. S. R. and Y. S.; visualization, S. S. R.; writing – original draft, S. S. R.; writing – reviewing and editing, M. P. C. M. and S. S. R.; funding acquisition, M. P. C. M.

## Conflicts of interest

There are no conflicts to declare.

## Supplementary Material

AY-016-D4AY00560K-s001
