# Supplementary material for: A (RP)UHPLC/UV analytical method to quantify dsRNA during the mRNA vaccine manufacturing process
Source: Anal Methods. 2024 Jul 8;16(30):5146–53. doi: 10.1039/d4ay00560k (PMC11293613; doi:10.1039/d4ay00560k)
Supplement: AY-016-D4AY00560K-s001 [file AY-016-D4AY00560K-s001.pdf]

Electronic Supplementary Material (ESI) for Analytical Methods.
This journal is © The Royal Society of Chemistry 2024

## Supplementary Material

# (RP)UHPLC/UV analytical method to quantify dsRNA during mRNA vaccine manufacturing process

Sara Sousa Rosa [a,b,c #], Shuran Zhang [a #], Yustika Sari [a], Marco PC Marques [a *]

[a] Department of Biochemical Engineering, University College London, Gordon Street, London,
WC1E 6BT, United Kingdom
[b] Department of Bioengineering, iBB—Institute for Bioengineering and Biosciences, Instituto Superior Técnico, Universidade de Lisboa, Lisboa, Portugal
[c] Associate Laboratory i4HB—Institute for Health and Bioeconomy, Instituto Superior Técnico, Universidade de Lisboa, Lisboa, Portugal

[#] These authors contributed equally to this work

*Correspondence:
**Dr. Marco P.C. Marques**
University College London, Biochemical Engineering, Bernard Katz Building, Gower Street, London, WC1E 6BT, United Kingdom
e-mail: marco.marques@ucl.ac.uk

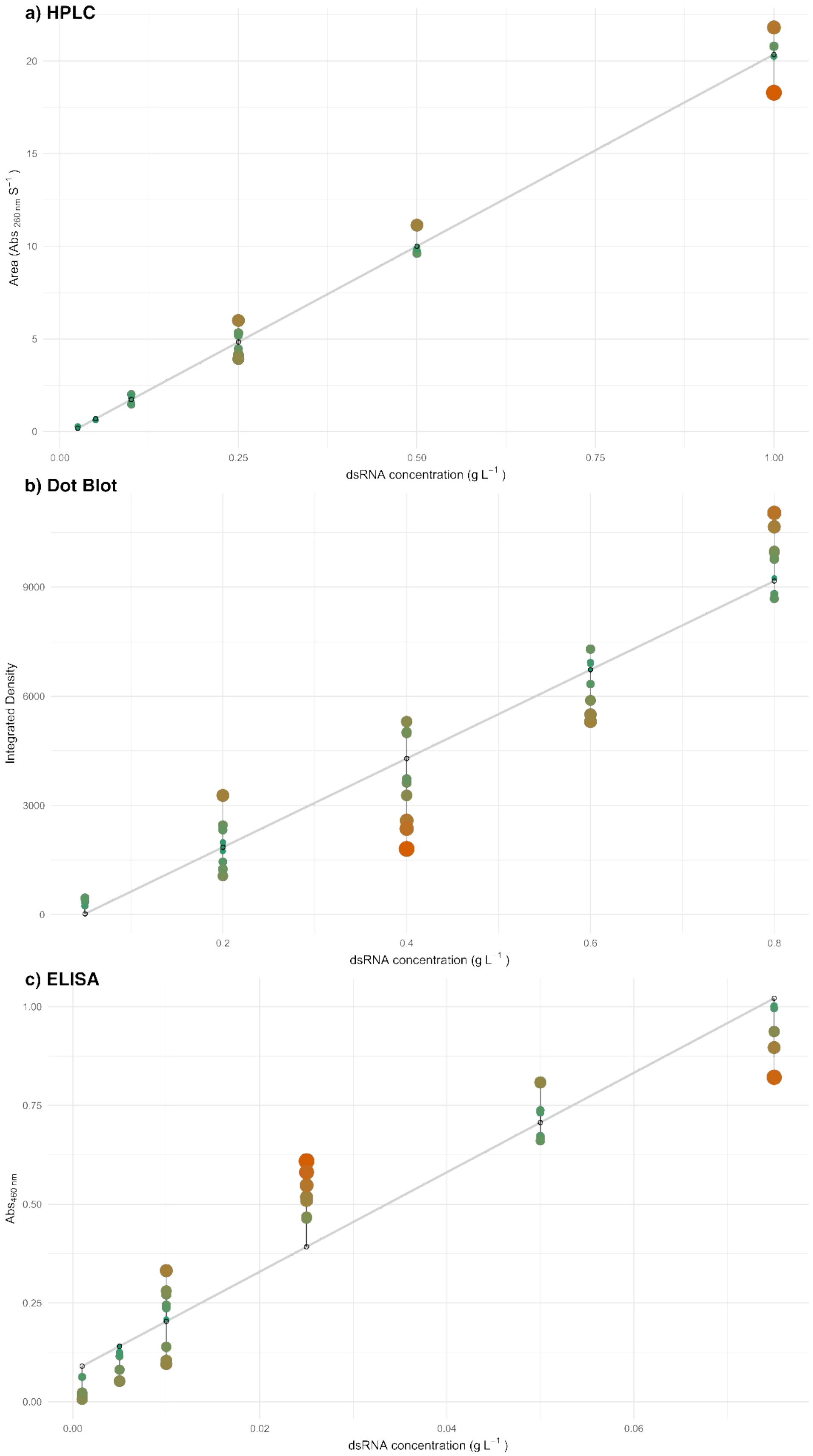


**Supplementary Figure S1**. Residual plots for the calibration curves used to calculated dsRNA concentration using the HPLC (a), the Dot Blot (b) and ELISA (c).

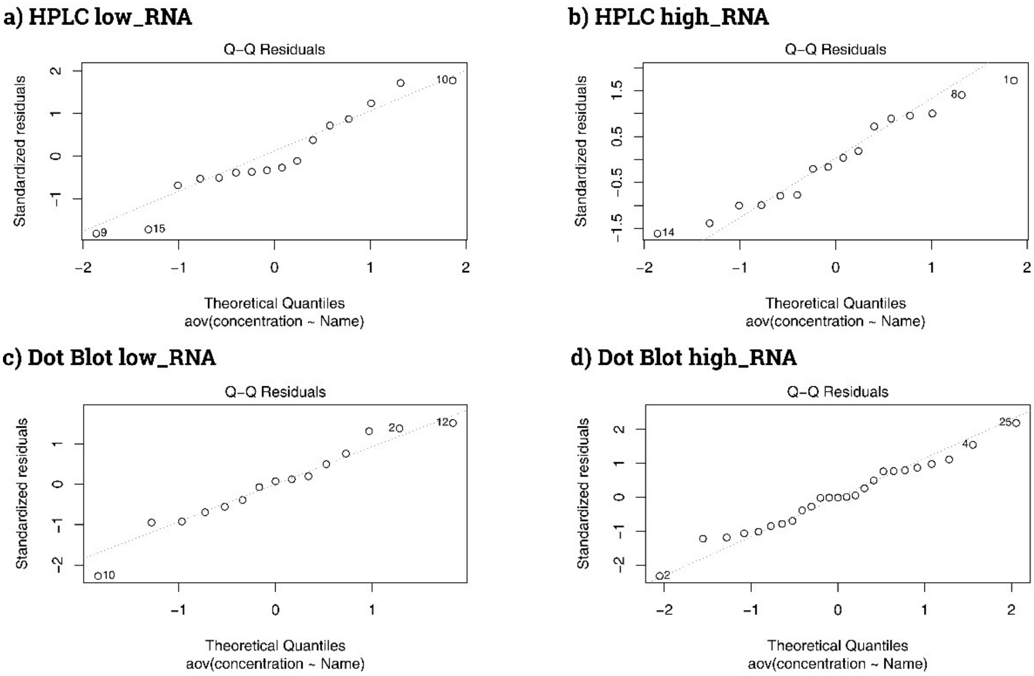


**Supplementary Figure S2**. Residual plots for the dsRNA spiking studies. Dot Blot results using 0.1 g $L^{-1}$ (a) and 0.4 g $L^{-1}$ dsRNA (b). HPLC results using 0.1 g $L^{-1}$ (c) and 0.4 g $L^{-1}$of dsRNA (d).

**Supplementary Table S1**. Dot Blot Tukey test results for high dsRNA concentration (0.4 g.$L^{-1}$). Concentration used for DNA correspond to 10 and 90 nM and to NTPs to 2 and 7.6 mM, for low and high, respectively.

| | | Confidence Interval at 95% | | | |
|---|---|---|---|---|---|
| Factor 1 | Factor 2 | Mean difference | Lower bound | Upper bound | Adjusted P value |
| **DNA_high** | DNA_low | 0.169 | -0.063 | 0.400 | 0.228 |
| | NTP_high | 0.319 | 0.108 | 0.531 | 0.002 |
| | NTP_low | 0.042 | -0.170 | 0.253 | 0.975 |
| | RNA_high | 0.008 | -0.212 | 0.228 | 1.000 |
| **DNA_low** | NTP_low | 0.151 | -0.061 | 0.362 | 0.246 |
| | DNA_low | -0.127 | -0.338 | 0.085 | 0.404 |
| | RNA_high | -0.161 | -0.381 | 0.059 | 0.223 |
| **NTP_low** | NTP_high | -0.277 | -0.467 | -0.088 | 0.002 |
| **RNA_high** | NTP_high | -0.312 | -0.510 | -0.113 | 0.001 |
| | NTP_low | -0.034 | -0.233 | 0.164 | 0.985 |

**Supplementary Table S2**. Dot BlotTukey test results for low dsRNA concentration. (0.1 $g.L^{-1}$). Concentration used for DNA correspond to 10 and 90 nM and to NTPs to 2 and 7.6 mM, for low and high, respectively.

| | | Confidence Interval at 95% | | | |
|---|---|---|---|---|---|
| Factor 1 | Factor 2 | Mean Difference | Lower bound | Upper bound | Adjusted P value |
| **DNA_high** | DNA_low | -0.004 | -0.052 | 0.044 | 0.998 |
| | NTP_high | -0.013 | -0.058 | 0.032 | 0.864 |
| | NTP_low | -0.022 | -0.076 | 0.031 | 0.663 |
| | RNA_low | -0.034 | -0.082 | 0.014 | 0.213 |
| **DNA_low** | NTP_low | -0.009 | -0.054 | 0.036 | 0.965 |
| | DNA_low | -0.018 | -0.071 | 0.036 | 0.808 |
| | RNA_low | -0.03 | -0.078 | 0.018 | 0.322 |
| **NTP_low** | NTP_high | -0.009 | -0.060 | 0.042 | 0.974 |
| **RNA_low** | NTP_high | -0.021 | -0.066 | 0.024 | 0.571 |
| | NTP_low | -0.012 | -0.065 | 0.042 | 0.947 |

**Supplementary Table S3**. HPLC Tukey test results for high dsRNA concentration (0.4 $g.L^{-1}$). Concentration used for DNA correspond to 10 and 90 nM and to NTPs to 2 and 7.6 mM, for low and high, respectively.

| | | Confidence Interval at 95% | | | |
|---|---|---|---|---|---|
| Factor 1 | Factor 2 | Mean Difference | Lower bound | Upper bound | Adjusted P value |
| **DNA_high** | DNA_low | -0.014 | -0.139 | 0.111 | 0.996 |
| | NTP_high | -0.041 | -0.157 | 0.075 | 0.775 |
| | NTP_low | 0.02 | -0.105 | 0.145 | 0.984 |
| | RNA_high | 0.014 | -0.129 | 0.156 | 0.998 |
| **DNA_low** | NTP_low | -0.028 | -0.153 | 0.098 | 0.950 |
| | DNA_low | 0.034 | -0.100 | 0.168 | 0.919 |
| | RNA_high | 0.027 | -0.122 | 0.177 | 0.974 |
| **NTP_low** | NTP_high | 0.061 | -0.064 | 0.187 | 0.534 |
| **RNA_high** | NTP_high | 0.055 | -0.087 | 0.197 | 0.724 |
| | NTP_low | -0.007 | -0.156 | 0.143 | 1.000 |

**Supplementary Table S4**. HPLC Tukey test results for low dsRNA concentration (0.1 g.L$^{-1}$). Concentration used for DNA correspond to 10 and 90 nM and to NTPs to 2 and 7.6 mM, for low and high, respectively.

| | | Confidence Interval at 95% | | | |
|---|---|---|---|---|---|
| Factor 1 | Factor 2 | Mean Difference | Lower bound | Upper bound | Adjusted P value |
| **DNA_high** | DNA_low | 0.011 | -0.011 | 0.033 | 0.503 |
| | NTP_high | -0.003 | -0.027 | 0.021 | 0.992 |
| | NTP_low | 0.007 | -0.017 | 0.031 | 0.874 |
| | RNA_low | 0.009 | -0.018 | 0.036 | 0.824 |
| **DNA_low** | NTP_low | -0.014 | -0.038 | 0.010 | 0.352 |
| | DNA_low | -0.004 | -0.028 | 0.020 | 0.976 |
| | RNA_low | -0.002 | -0.029 | 0.025 | 0.998 |
| **NTP_low** | NTP_high | 0.010 | -0.015 | 0.036 | 0.710 |
| **RNA_low** | NTP_high | 0.012 | -0.017 | 0.040 | 0.666 |
| | NTP_low | 0.002 | -0.027 | 0.030 | 0.999 |
